# Therapeutic effects of NAC and CoQ10 on aluminium phosphide poisoning as an adjuvant therapy: A Pilot study

**DOI:** 10.1016/j.toxrep.2025.101907

**Published:** 2025-01-21

**Authors:** Maii Farag Henaidy, Maha Ghanem, Shehata Farag Shehata, Amal M. Shouair, Reda R. Mabrouk

**Affiliations:** a Department of Forensic Medicine and Clinical Toxicology, Faculty of Medicine, Alexandria University, Egypt; b Department of Family and Community Medicine, King Khalid University, Abha, Saudi Arabia; c High Institute of Public Health, Alexandria University, Egypt; d Directorate of Health Affairs in Buhaira-Clinical Research Department, Ministry of Health and Population, Damanhour, Egypt; e Pharmaceutical Medicinal Chemistry& Drug Design Department, Faculty of Pharmacy (Boys), Al-Azhar University, Egypt

**Keywords:** Humans, Length of stay, Aluminium phosphide, Poisons, Coenzyme Q10, Suicide

## Abstract

In aluminium phosphide (AlP) poisoning, death is mainly due to acute heart failure. There is some evidence showing that N-acetylcysteine (NAC) and coenzyme Q10 have antioxidant and cardioprotective effects. This study investigated a new approach for treating acute AlP poisoning by using NAC and Co-Q10 as adjuvant therapy.

**Subjects and methods:**

The study design was a retrospective-prospective study. It was conducted in the poisoning unit of Kafer Eldwar General Hospital. Sixty patients with acute aluminium phosphide poisoning were included. The patients were divided into two groups. The first group (standard protocol) considered the control group received the standard supportive care, and their data were collected from the medical records. The second group (new protocol) in addition to the standard supportive care received the NAC and CoQ10 regimen, and all data were collected in a specially designed sheet.

**Results:**

The results showed that the highest percentage of patients in both groups were aged 18–25, followed by those under 18, and females outnumbered the males. The systolic (SBP) and diastolic (DBP) blood pressure showed significant improvement in the new protocol group. A significant statistical difference was found between the two groups regarding mechanical ventilation (p = 0.015), where mechanical ventilation was used in 20 % of patients in the new protocol group and 50 % in the standard group. Regarding the outcome of patients, the survival rate reached 73.3 % upon using the new protocol, compared to 50 % who received the standard protocol.

**Conclusion:**

The data imply that further investigation in using the NAC and CoQ10 regimen is warranted. It gave an improvement of the survival rate and decrease the need for mechanical ventilation in AlP

## Highlight

• NAC and co-Q10 were significantly improving the outcome in new protocol in treatment of AlP intoxication.
• There's a significant correlation between new protocol treatment with the need to mechanical ventilation and survival rate.
• The new protocol group showed significant improvement in the systolic and diastolic blood pressure.

## Graphical Abstract

**Figure:**
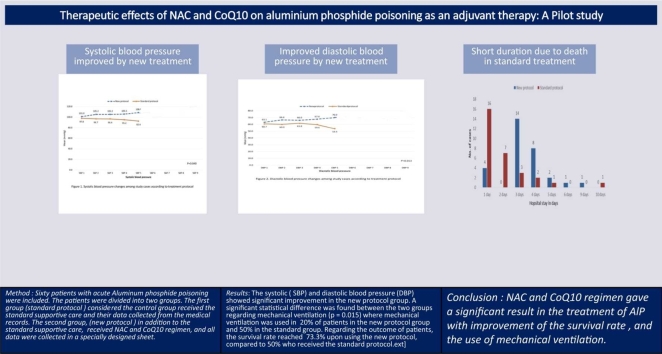

## 1 Introduction

AlP poisoning remains a significant public health issue, particularly in regions where it is used for agricultural purposes. Suicidal ingestion is the primary cause of AlP poisoning, though accidental exposure can occur. This pesticide is not only toxic to the environment but also poses a serious threat to human health [1], [2].

The preferred substance in a suicide attempt in some countries, e.g., Egypt, Mexico, and Iran, is aluminium phosphide, as it is cheap and easy to use. In research, 35 percent of adolescent suicides involved AlP use [3], [4], [5]. The toxic effects of AlP are due to deadly phosphine gas liberated when it reacts with water or hydrochloric acid in the stomach. In the case of oral intake, the phosphine gas released is absorbed by the gastrointestinal tract with simple diffusion and is excreted by the kidneys and lungs [6].

On the cellular level, mitochondria are abundant in cardiomyocytes, and by producing ATP through the process of oxidative phosphorylation, they contribute to the contractile function of cardiomyocytes and provide 90 % of the energy of these cardiomyocytes [7].

Phosphine inhibits cytochrome c oxidase and the mitochondrial respiratory chain enzymes. Hence, it leads to the generation of reactive oxygen species. Phosphine also has been shown to increase the generation of reactive oxygen species (ROS) and promote oxidative stress by inhibiting enzymatic antioxidants such as catalase (CAT), glutathione, glutathione reductase (GR), and superoxide dismutase (SOD). These changes accelerate lipid peroxidation, resulting in cell membrane damage, ionic barrier disruption, nucleic acid damage, and cell death. AlP produced reactive oxygen species (ROS) due to mitochondrial dysfunction. ROS production leads to red blood cell hemolysis, decreased ATP production, and induction of apoptosis in cardiomyocytes, which eventually results in cardiovascular disease. Since ALP has the most significant effect on cardiomyocytes, the use of appropriate treatment strategies to restore cell function can increase patients’ survival [8], [9], [10].

Drug targeting to the subcellular compartments represents one of the modern trends in molecular pharmacology. Although the approach for targeting mitochondria was developed 50 years ago, it only became widely used in the last decade [11].

Mitochondrial dysfunction plays a crucial role in the pathogenesis of AlP poisoning. Recent research has focused on targeting mitochondria to alleviate the toxic effects of AlP. One promising agent is CoQ10, a powerful antioxidant that can improve mitochondrial function and reduce oxidative stress. By targeting mitochondrial dysfunction, CoQ10 may offer a novel therapeutic approach to treat AlP poisoning [11], [12].

NAC is an important antioxidant and cytoprotective agent that replenishes intracellular glutathione in preclinical trials. NAC has been shown to have a protective role against cardiovascular complications by protecting heart cells from the oxidative stress induced by phosphine [13], [14].

The combination of NAC and coenzyme CoQ10 showed that the antioxidants have a remarkable cardioprotective effect, suggesting that they may be useful as prophylactic agents against the detrimental effects of cardiotoxins [15].

The use of two drugs, N-acetylcysteine (NAC) and coenzyme Q10 (CoQ10), in pregnant women contributes to the patient's stabilization. This combination played a crucial role in ensuring the well-being of both the pregnant woman and the foetus [16].

Based on the experience of the author in a case report, this study investigated a new approach for treating acute AlP poisoning using NAC and CoQ10 as adjuvant therapy.

## 2 Study design and setting

The study design was a retrospective-prospective study (also called a hybrid design). Control group: Data were obtained retrospectively from existing records. Intervention group: Data were collected prospectively through active assessment. This hybrid approach combines elements of retrospective and prospective study designs, allowing researchers to analyze historical data while simultaneously collecting real-time data for comparison. It is commonly used when existing data are available for one group but new data needs to be collected from another group [17].

All patients (60) with acute aluminium phosphide poisoning admitted in a period between 1st December 2022 and the end of August 2024 were included.

### 2.1 Inclusion criteria

AIP-poisoned patients aged 14 and older were included in this study. Inclusion criteria were acidosis and a systolic blood pressure (SBP) below 100 mmHg.

### 2.2 Exclusion criteria

All the following patients were excluded: inhaled AlP, ingested expired AlP tablets, those who received any treatment before admission, patients with other medical problems such as heart diseases, hepatic or renal disease, patients with immune-compromised conditions, co-ingestion of other material, refusal to participate in the trial, hypersensitivity to NAC or CoQ10, and acute myocardial infarction.

### 2.3 Data collection and analysis

This study collected data from Kafer Eldwar General Hospital. The variables analyzed included gender, age, route of administration, type of poisoning, length of hospital stay, and patient outcome.

The collected data were subjected to statistical analysis and tabulation using the SPSS program, version 20. The chi-square test was used to test the association between variables; we considered P ≤ 0.05 statistically significant [18], [19].

All data were found to be normally distributed.

### 2.4 Ethical considerations

The Institutional Review Board, Ministry of Health and Population, Egypt, approved the study (IRB Number: 0000687, FWA Number: 00016183, Serial Protocol Number: 0005704). Informed consent was taken from the patients or their relatives.

## 3 Method

Sixty patients with acute aluminium phosphide poisoning were included. The patients were divided into two groups.

**The first group** (standard protocol), considered a control group, received the standard supportive care, and their data was collected from the medical records.

The second group (new protocol) received the standard supportive care and received the NAC and CoQ10 regimen. Data was collected in a specially designed sheet. They were subjected to:

A- Detailed history taking (personal and medical, AlP exposure data).

B. Thorough clinical assessment, vital signs, and general examination.

C-Laboratory investigations that included arterial blood gases (ABG), complete blood count, serum sodium and potassium levels, liver enzymes, and cardiac enzymes (CK-MB, troponin).

D- Electrocardiogram (ECG) and electrocardiograph

Vital signs were assessed at admission and 6, 12, 18, and 24 h post-admission. (5 times)

### 3.1 Treatment

**Standard protocol group:** the patients received the standard supportive care, including - Care of GIT decontamination using paraffin oil Paraffin oil: dose 200–250 ml, Children 50–100

– Sodium bicarbonate: 1–2 meq/kg and repeated if needed
– Care of airway (inhalation of 100 % oxygen),
– Normal saline solution: Saline is given 500 ml/12 h
– Intravenous noradrenalin: (4 mg/2 ml) in 50 ml glucose 5 % (0.01–3 mcg/kg/min)

The second group (**New protocol group):** In addition to the standard supportive care, a dual therapy approach was adopted to address ALP toxicity.

NAC regimen was administered at a dose of 150 mg/kg intravenously over 1 h, followed by 50 mg/kg over 4 h, and then 100 mg/kg over 16 h in 5 % dextrose.

Furthermore, patients received 300 mg of CoQ10 as an antioxidant therapy, dissolved in the paraffin oil. Subsequently, CoQ10 was continued at a dose of 200 mg/day every 12 h [12], [16].

2. All the patients were followed up until discharge from the hospital or death.

3. The outcome, hospital stay, mortality rate, and ICU admission were recorded.

### 3.2 Safety assessment of NAC and coenzyme Q10

Ikematsu et al. [20] investigated the safety of high-dose coenzyme Q10 in healthy adults. Participants received 300, 600, or 900 mg of Q10 daily for four weeks. No serious adverse events were reported in any group. Mild side effects, such as common cold symptoms and gastrointestinal issues, were observed but were not dose-dependent and considered unrelated to Q10.No clinically significant changes were observed in hematology, blood biochemistry, or urinalysis. In conclusion, Q10 was well-tolerated and safe in healthy adults at doses up to 900 mg/day.

Tenório et al. [21] stated that NAC, used in both oral and intravenous forms, generally has a good safety profile. In I.V. administration, it can cause nausea and vomiting (up to 9 %). Serious adverse effects are rare but possible, including anaphylactoid reactions (up to 8.2 %).

## 4 Results

Sixty acute aluminium phosphide-intoxicated patients were included in the study that met the eligibility criteria. The patients were admitted to the hospital between 1st December 2022 and the end of August 2024.

### 4.1 Age, sex, and number of ingested tablets

Table 1 shows that the highest percentage of patients in both groups were aged 18–25 (36.7 % and 40 %, respectively), followed by those under 18 (33.3 %). Regarding sex, 50 % of patients in the standard protocol group and 60 % in the new protocol group were female. Most patients in both groups took only one tablet (83.3 % and 96.7 %, respectively).

**Table 1:** Bio-demographic characteristics among study groups.

**Variable**	**Group**				**p-value**
	**Group2 New protocol (n = 30)**		**Group1 Standard protocol (n = 30)**		
	**No**	**%**	**No**	**%**	
**Age in years**					.950
< 18	10	33.3 %	10	33.3 %	
18–24	11	36.7 %	12	40.0 %	
25 +	9	30.0 %	8	26.7 %	
Mean ± SD		24.1 ± 11.8	22.6 ± 9.5		
**Gender**					.436
Male	15	50.0 %	12	40.0 %	
Female	15	50.0 %	18	60.0 %	
**Number of tablets**					.304^
.25	2	6.7 %	0	0.0 %	
.50	2	6.7 %	1	3.3 %	
1.00	25	83.3 %	29	96.7 %	
2.00	1	3.3 %	0	0.0 %	
**Immediate vomiting after ingestion**					.001*
Yes	22	73.3 %	8	26.7 %	
No	8	26.7 %	22	73.3 %	
**GCS**					.059$
Range	3–15		3–15		
Mean ± SD	13.8 ± 3.5		12.0 ± 3.8		
**The time interval between onset of toxicity and beginning of treatment (minutes)**					.095$
Range	30–240		20–240		
Mean ± SD	89.8 ± 49.9		117.7 ± 74.3		
Median	60		120		

P: Pearson X^2^ test.

$ Independent *t*-test.

^ Exact probability test.

* *P* < 0.05 (significant).

### 4.2 ECG and acidosis

An electrocardiogram (ECG) was performed on all patients upon admission to assess cardiac function. The majority of the patients presented with normal sinus rhythm (93.3 % and 96.7 %, respectively). Less than half of the patients in both groups received sodium bicarbonate (NaHCO₃) (43.3 % in the standard protocol group and 40 % in the new protocol group). (Table 2)

**Table 2:** Medical intervention and investigations among study groups.

**Variable**	**Group**				**p-value**
	**Group2 New protocol** (**n = 30)**		**Group1 Standard protocol (n = 30)**		
	**No**	**%**	**No**	**%**	
**ECG**					.221
Bradycardia	2	6.7 %	0	0.0 %	
Flat	0	0.0 %	1	3.3 %	
Normal	28	93.3 %	29	96.7 %	
**NaHCO3**					.793
Yes	12	40.0 %	13	43.3 %	
No	18	60.0 %	17	56.7 %	
**Vasopressor**					.559
Noradrenaline Infusion	7	23.3 %	9	30.0 %	
Not given	23	76.7 %	21	70.0 %	
**Use of MV**					.015*
Yes	6	20.0 %	15	50.0 %	
No	24	80.0 %	15	50.0 %	

P: Exact probability test.

* *P* < 0.05 (significant).

### 4.3 Vital signs

#### 4.3.1 Vital signs were assessed at admission and 6, 12, 18, and 24 h post-admission

**Fig. 1:**
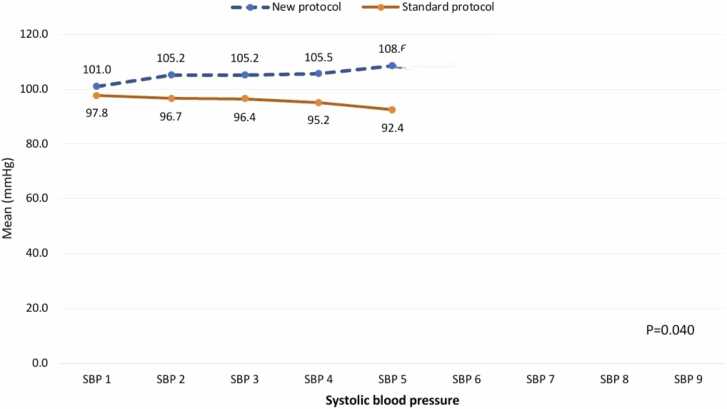
Systolic blood pressure changes among study cases according to treatment protocol.

Fig. 1 shows the systolic blood pressure (SBP) values at different time points for the "new protocol" and "standard protocol" groups. The "new protocol" group generally had higher SBP values than the "standard protocol" group. At SBP_1, the "new protocol" group had a mean SBP of 101.03, compared to 97.78 in the "standard protocol" group. This pattern of higher SBP in the "new protocol" group continued through the study, with values ranging from 104.82 to 110.87, while the "standard protocol" group’s SBP values ranged from 92.39 to 97.78. The most reported difference was at SBP_5, where the "new protocol" group had a mean SBP of 108.57, significantly higher than the 92.39 in the "standard protocol" group, showing a difference of 16.18 mmHg. Differences between the two groups were smaller at time points like SBP_2 (105.17 vs. 96.67) and SBP_3 (105.17 vs. 96.40), but the "new protocol" group still had higher readings at each time point. The difference between the groups was statistically significant (p = 0.040).

**Fig. 2:**
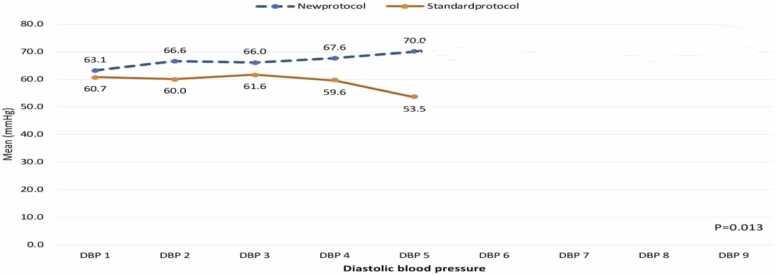
Diastolic blood pressure changes among study cases according to treatment protocol.

Fig. 2 illustrates the diastolic blood pressure (DBP) values at different time points for the "new protocol" and "standard protocol" groups, showing a consistent trend of higher DBP in the "new protocol" group. At DBP_1, the mean DBP in the "new protocol" group was 63.10 mmHg, slightly higher than the 60.74 mmHg observed in the "standard protocol" group. This small difference continued at DBP_2, where the "new protocol" group had a mean DBP of 66.55 mmHg compared to 60.00 mmHg in the "standard protocol" group, marking a difference of 6.55 mmHg. By DBP_3, the gap remained at 4.43 mmHg, with the "new protocol" group showing a mean DBP of 66.03 mmHg versus 61.60 mmHg in the "standard protocol" group. The difference increased at DBP_4, where the "new protocol" group had a mean DBP of 67.59 mmHg, compared to 59.57 mmHg in the "standard protocol" group, reflecting an 8.02 mmHg difference. The most significant gap appeared at DBP_5, with the "new protocol" group showing a mean DBP of 70.00 mmHg, compared to just 53.48 mmHg in the "standard protocol" group, resulting in a difference of 16.52 mmHg. These differences were statistically significant (p = 0.013), indicating that the "new protocol" group consistently exhibited higher diastolic blood pressure across all time points, with the largest difference observed at DBP_5.

### 4.4 Treatment and mechanical ventilation

Table 2 also shows that more than two-thirds of patients in both groups received noradrenaline infusion. Mechanical ventilation was used in 20 % of patients in group 2, and 50 % in group 1 required mechanical ventilation. A significant statistical difference was found between the two groups regarding mechanical ventilation (p = 0.015).

### 4.5 Duration of hospitalization

**Fig. 3:**
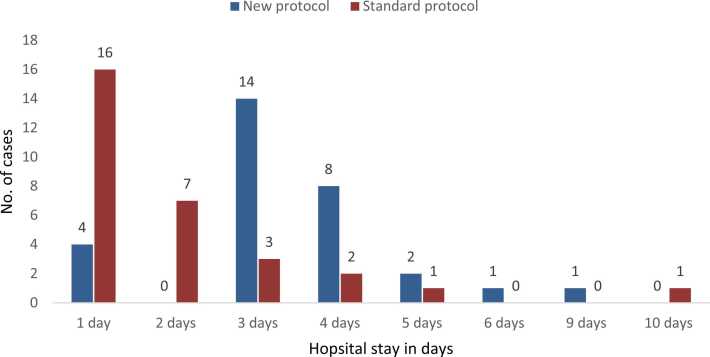
Hospital stay among study cases by their protocol of treatment.

Fig. 3 illustrates the duration of hospitalization for patients in the "new protocol" and "standard protocol" groups, highlighting some notable differences between the two. In the "new protocol" group, most patients (46.7 %) were hospitalized for 3 days, followed by 26.7 % who stayed for 4 days and 13.3 % who were discharged after just 1 day. In contrast, the "standard protocol" group had a significantly higher percentage of patients discharged after only 1 day (53.3 %), with 23.3 % remaining for 2 days. Only 10 % of patients in the "standard protocol" group were hospitalized for 3 days, and even fewer stayed longer. These findings suggest that patients in the "new protocol" group experienced slightly longer hospital stays, with most patients remaining between 3 and 6 days. Conversely, the "standard protocol" group appeared to have quicker recoveries, with more patients discharged within 1 or 2 days.

### 4.6 Outcomes of patients

Table 3 displays the outcomes of patients in the "new protocol" and "standard protocol" groups. In the "new protocol" group, 8 patients (26.7 %) died, compared to 15 patients (50.0 %) in the "standard protocol" group (p-value = 0.043), indicating that this difference is statistically significant, suggesting that the "new protocol" may have had a positive effect on survival. Looking at the time between taking the antidote and death, the "new protocol" group had a mix of outcomes: 25 % of patients died within 2 h, another 25 % within 4 h, and 50 % survived for at least 24 h after receiving the antidote.

**Table 3:** Outcome of patients by their treatment protocol.

**Outcome**	**Group**				**p-value**
	**Group2 New protocol (n = 30)**		**Group1 Standard protocol (n = 30)**		
	**No**	**%**	**No**	**%**	
**Death**					.043*
Died	8	26.7 %	15	50.0 %	
Survived	22	73.3 %	15	50.0 %	

P: Pearson X^2^ test

* *P* < 0.05 (significant).

## 5 Discussion

The primary mechanism of AlP poisoning is not fully understood; hence, no specific antidote was recommended. Numerous investigations have shown that oxidative stress and disruptions in the electron transport chain play a major role in the pathophysiology of AlP poisoning. The suggested mechanism of ALP toxicity includes the inhibition of oxidative phosphorylation and the cytochrome-c oxidase enzyme, leading to a breakdown in cellular respiration and generating oxidative stress that harms cells through lipid damage. Thus, antioxidant treatment could offer therapeutic advantages in cases of acute ALP poisoning [22], [23].

In the present study, females outnumbered males, as they represented 60 % of the new treatment protocol group. The first group (standard treatment) data were chosen equally for statistical consideration. High levels of stress on the emotional, financial, and intellectual fronts, failure to meet goals, and easy availability of poisons are all possible causes of the high percentage of suicidal poisoning during these adults' fruitful years. In several studies, females have markedly outnumbered males all through the years in different countries such as Sri Lanka, Saudi Arabia, and Egypt [24], [25], [26].

In this clinical study, we focused on specific patient data, including metabolic acidosis, ECG readings, and blood pressure. We were particularly interested in how these factors related to outcomes such as the need for mechanical ventilation, length of hospital stay, and mortality rate.

Less than half of the patients in both groups received sodium bicarbonate (NaHCO₃) with no significant statistical results. Metabolic acidosis is a common complication resulting from AlP poisoning. This condition arises from circulatory failure and inadequate tissue perfusion. Individuals with AlP poisoning might experience metabolic acidosis that could be persistent and resistant to typical treatment methods. The conventional approach to metabolic acidosis is intravenous HCO3, but in cases of severe and unresponsive metabolic acidosis, hemolysis may be required [27]. On the contrary, Darwish et al. [12] mentioned a decrease in acidosis with Q10 administration.

For cardiovascular assessment, an electrocardiogram (ECG) was performed on all patients. Over 90 % of patients in both groups exhibited a normal heart rhythm. The majority presented with normal sinus rhythm, as expected due to early hospital admission.

Vital signs were assessed at admission and 6, 12, 18, and 24 h post-admission.

Both groups exhibited similar patterns of vasopressor use, with no significant difference observed. Minor variations were noted in the new treatment group.

Both systolic blood pressure (SBP) and diastolic blood pressure (DBP) were significantly higher in the new protocol group compared to the standard protocol group across all five measurements.

NAC and co-Q10 treatment successfully improved cardiogenic shock and elevated SBP levels. These findings align with a study by Taghaddosinejad et al. [28] in Iran, which demonstrated significant improvements in both SBP and DBP in 63 AIP patients over 24 h (0, 12, 18, and 24 h). Furthermore, Darwish et al. [12] reported a similar increase in blood pressure following Q10 administration.

A significant reduction in using mechanical ventilation was seen between the two groups (p = 0.015). A similar reduction in using mechanical ventilation was mentioned in Taghaddosinejad et al. [28] and Shaker et al. [23], where both noticed the decreased need for mechanical ventilation in using NAC [23], [28].

The apparent significant decrease in hospital stay in the standard protocol group appeared in one to two days due to an increased mortality rate, which led to a shortening of hospitalization for these patients.

The new treatment protocol was associated with a significant reduction in mortality rate, aligning with the findings of Shaker et al. [23]. Their study demonstrated that intravenous N-acetylcysteine can effectively reduce mortality in severe aluminium phosphide poisoning cases. In a cohort of 91 patients, mortality rates were 43.95 % in the N-acetylcysteine group and 66.27 % in the control group.

Other studies by Tehrani et al. [14], El Ebiary et al. [29], and Emam et al. [30] confirm these results. The process of oxidative stress and the depletion of reduced glutathione lead to an increase in total antioxidant capacity (TAC) levels. Considering these facts in their studies, increased serum levels of malondialdehyde (MDA) and TAC in ALP-intoxicated patients relative to the reference values were observed as markers for oxidative stress induced by ALP.

Based on oxidative stress theory in acute ALP poisoning, coenzyme Q10 (CoQ10) was examined for a probable cardioprotective role in ALP poisoning, focusing on its antioxidant capacity. Several studies mentioned that CoQ10 has a cardioprotective effect and ameliorates ALP-induced cardiotoxicity [31], [32].

To our knowledge, no study used the combined effects of NAC and Q10. Except for the case study by one of the authors [16], as mentioned before, the reduction in mortality rate indicates successful management in the new protocol group.

In conclusion, the data imply that further investigation in using the NAC and CoQ10 regimen is warranted. It gave an improvement of the survival rate and decrease the need for mechanical ventilation in Alp.

### 5.1 Limitation of the study

It is difficult to recruit a sufficient sample size for a clinical trial within a reasonable timeframe. Small sample sizes can introduce several limitations, including the risk of random variability, lack of precision and reliability, and limited exploration of heterogeneity. We tried to compare our results with other large studies, but unfortunately, no similar studies were met. So, we tried to adjust the independent variables in the final analysis, stratifying the patients, using pairwise comparisons, and using statistical techniques that match the anticipated response curve.

We recommend conducting future research with larger sample sizes to confirm your findings and provide more robust conclusions.

## Fund statement

No fund

## Author statement

We the undersigned declare that this manuscript is original, has not been published before and is not currently being considered for publication elsewhere. We confirm that the manuscript has been read and approved by all named authors and that there are no other persons who satisfied the criteria for authorship but are not listed. We further confirm that the order of authors listed in the manuscript has been approved by all of us. We understand that the Corresponding Author is the sole contact for the Editorial process. She is responsible for communicating with the other authors about progress, submissions of revisions and final approval of proofs.

## CRediT authorship contribution statement

**Maha Ghanem:** Writing – review & editing, Writing – original draft, Supervision. **Maii Farag Henaidy:** Methodology, Conceptualization. **Reda R. Mabrouk:** Validation, Software. **Shehata Farag Shehata:** Formal analysis. **Amal M. Shouair:** Investigation.

## Declaration of Competing Interest

The authors declare that they have no known competing financial interests or personal relationships that could have appeared to influence the work reported in this paper.

## Data Availability

Data will be made available on request.

## References

[1] Sobh Z., Ghanem M., Kholief M. (2023). Physicians’ perspectives on different therapeutic approaches for aluminium phosphide poisoning and their relevant outcomes. Toxicol. Res..

[2] Malakar S., Negi B.D., Dutt K., Raina S. (2019). Intravascular haemolysis in aluminium phosphide poisoning. Indian J. Crit. Care Med..

[3] Ghanem M.A., Sultan E.A., Gaber Mahmoud H.R., Ahmed Raafat O.M. (2021). Adolescent’s suicide using pesticides: risk factors and outcome prediction. Asia Pac. J. Med. Toxicol..

[4] Reyna-Medina M., Vázquez-de Anda G.F., García-Monroy J., Valdespino-Salinas E.A., Vicente-Cruz D.C. (2013). Tentative suicida por intoxicación con fosfuro de aluminio [Suicide attempt with aluminium phosphide poisoning]. Rev. Med. Inst. Mex. Seguro Soc..

[5] Etemadi-Aleagha Afshar M.D., Akhgari Maryam Pharm D., Ph.D., Iravani Fariba Sardari M.D. (2015). Aluminium phosphide poisoning-related deaths in Tehran, Iran, 2006 to 2013. Medicine.

[6] Meena M.C., Mittal S., Rani Y. (2015). Fatal aluminium phosphide poisoning. Inter. Toxicol..

[7] Li A., Shami G.J., Griffiths L., Lal S., Irving H., Braet F. (2023). Giant mitochondria in cardiomyocytes: cellular architecture in health and disease. Basic Res. Cardiol..

[8] Hosseini S.F., Forouzesh M., Maleknia M., Valiyari S., Maniati M., Samimi A. The Molecular Mechanism of Aluminium Phosphide poisoning in Cardiovascular Disease: Pathophysiology and Diagnostic Approach. Cardiovasc Toxicol. 2020 Oct;20(5):454-461. doi: 10.1007/s12012-020-09592-4. Erratum in: Cardiovasc Toxicol. 2020 Oct;20(5):462. doi: 10.1007/s12012-020-09604-3. PMID: 32712815. 10.1007/s12012-020-09592-4 32712815

[9] Isackson B., Irizarry L. Rodenticide Toxicity. [Updated 2024 Jun 22]. In: StatPearls [Internet]. Treasure Island (FL): StatPearls Publishing, 2024 Jan-. Available from: 〈https://www.ncbi.nlm.nih.gov/books/NBK554428/〉. 32119315

[10] Baghaei A., Solgi R., Jafari A., Abdolghaffari A.H., Golaghaei A., Asghari M.H., Baeeri M., Ostad S.N., Sharifzadeh M., Abdollahi M. (2016). Molecular and biochemical evidence on the protection of cardiomyocytes from phosphine-induced oxidative stress, mitochondrial dysfunction, and apoptosis by acetyl-L-carnitine. Environ. Toxicol. Pharmacol..

[11] Zinovkin R.A., Zamyatnin A.A. (2019). Mitochondria-targeted drugs. Curr. Mol. Pharmacol..

[12] Darwish R.T., Sobh Z.K., Hamouda E.H., Saleh E.M. (2020). The efficacy of Coenzyme Q10 and liquid paraffin oil in the management of acute aluminium phosphide poisoning. Toxicol. Res..

[13] Shakeri S., Mehrpour O. (2014). Aluminium phosphide poisoning in animals. Int. J. Med. Toxicol. Forensic Med..

[14] Tehrani H., Halvaie Z., Shadnia S., Soltaninejad K., Abdollahi M. (2013). Protective effects of N-acetylcysteine on aluminium phosphide-induced oxidative stress in acute human poisoning. Clin. Toxicol..

[15] Elbaky N.A.A., El-Orabi N.F., Fadda L.M., Abd-Elkader O.H., Ali H.M. (2018). Role of N-Acetylcysteine and Coenzyme Q10 in the amelioration of myocardial energy expenditure and oxidative stress, induced by carbon tetrachloride intoxication in rats. Dose Response.

[16] Henaidy M., Noah a, El-zahabi M., Gemeah M., Shouair M., Mabrouk R. (2024). Effective management of acute aluminium phosphide poisoning in pregnancy: a case study of dual therapy efficacy and collaborative care. J. Med. Res. Inst..

[17] Rothman K.J., Greenland S., Lash T.L. (2008). Modern Epidemiology.

[18] IBM SPSS Statistics for Windows, Version 20.0. Armonk, NY: IBM Corporation, 2012. 11.

[19] Kirkpatrick L.A., Feeney B.C. (2013).

[20] Ikematsu H., Nakamura K., Harashima S., Fujii K., Fukutomi N. (2006). Safety assessment of coenzyme Q10 (Kaneka Q10) in healthy subjects: a double-blind, randomized, placebo-controlled trial. Regul. Toxicol. Pharmacol..

[21] Tenório M.C.D.S., Graciliano N.G., Moura F.A., Oliveira A.C.M., Goulart M.O.F. (2021). N-Acetylcysteine (NAC): impacts on human health. Antioxidants.

[22] Goharbari M., Taghaddosinejad F., Arefi M. (2018). Therapeutic effects of oral liothyronine on aluminium phosphide poisoning as an adjuvant therapy: a clinical trial. Hum. Exp. Toxicol..

[23] Shaker H.O., Rageh O.E.S., Alnajar M., Alshamaly N.F., Abdelmaged W.A., Abd-ElGawad M. (2023). Efficacy of intravenous N acetylcysteine as an adjuvant therapy in the treatment of acute aluminium phosphide Poisoning: a systematic review and meta-analysis. BMC Pharmacol. Toxicol..

[24] Peshin S.S., Gupta Y.K. (2018). Poisoning due to household products: a ten years retrospective analysis of telephone calls to the National Poisons Information Centre, All India Institute of Medical Sciences, NewDelhi, India. J. Forensic Leg. Med..

[25] Abdelhamid W. (2021). Evaluation of severity of poisoning exposures among patients presented to poison control center, Ain Shams University Hospitals, Egypt during 2019. Ain Shams J. Forensic Med. Clin. Toxicol..

[26] Aggarwal N., Sawlani K.K., Chaudhary S.C., Usman K., Dandu H., Atam V. (2023). Study of pattern and outcome of acute poisoning cases at tertiary care hospital in North India. J. Fam. Med. Prim. Care.

[27] Menzies K.J., Robinson B.H., Hood D.A. (2009). Effect of thyroid hormone on mitochondrial properties and oxidative stress in cells from patients with mtDNA defects. Am. J. Physiol. Cell Physiol..

[28] Taghaddosinejad F., Farzaneh E., Ghazanfari-Nasrabad M., Eizadi-Mood N., Hajihosseini M., Mehrpour O. (2016). The effect of N-acetyl cysteine (NAC) on aluminium phosphide poisoning inducing cardiovascular toxicity: a case-control study. Springerplus.

[29] El-Ebiary A., Abuelfad A. (2017). N-acetylcysteine as an adjuvant in the treatment of acute aluminium phosphide poisoning: a randomized clinical trial. Ain Shams J. Forensic Med. Clin. Toxicol..

[30] Emam N., Shaaban S., Ahmed D., Could N., Could N. (2020). Acetyl cysteine prolong survival time in acute aluminium phosphide poisoning among egyptian patients?. Egypt. Soc. Clin. Toxicol. J..

[31] Elsharkawy Rasha E., Ghonem Mona M., El-Sarnagawy Ghada N., Nagy Ayman A., Heshmat Mona M. (2023). Cardioprotective role of the coenzyme Q10 and coconut oil in acute aluminium phosphide poisoning: a randomized controlled clinical trial. Toxicol. Res..

[32] Hooshangi Shayesteh M.R., Hami Z., Chamanara M., Parvizi M.R., Golaghaei A., Nassireslami E. (2024). Evaluation of the protective effect of coenzyme Q10 on hepatotoxicity caused by acute phosphine poisoning. Int. J. Immunopathol. Pharmacol..

